# *Plasmodium* Parasitemia and Its Relationship With Hematological, Liver, and Kidney Function of Patients With *Falciparum* Malaria in the Eastern Region, Ghana

**DOI:** 10.1002/hsr2.72085

**Published:** 2026-03-11

**Authors:** Augustine Badu, Isaac Darban, Kofi Mensah, Maxwell Hubert Antwi, Mark Appeaning, Aaron Siaw Kwakye, Tonnies Abeku Buckman, Vidic Kwarteng Owusu, Daniel Takyi Mintah, Raymond Ansah Agyei, Comfort Yeboah, Daniel Acorlor

**Affiliations:** ^1^ Department of Medical Laboratory Science Koforidua Technical University Koforidua Ghana; ^2^ Department of Medical Laboratory Science University of Development Studies Ghana; ^3^ Department of Medical Laboratory Science KAAF University College Buduburam Ghana; ^4^ Laboratory Department Medicas Hospital Mampong‐Akuapem Ghana; ^5^ Laboratory Department New Abirim Government Hospital New Abirem Eastern Region Ghana; ^6^ Global Evangelical Mission Hospital KNUST Ghana; ^7^ Municipal Health Directorate Upper Denkyira East Dunkwa on Offin Ghana

**Keywords:** adults, children, Ghana, parasitemia, *Plasmodium*

## Abstract

**Background:**

Malaria remains a significant public health challenge in Sub‐Saharan Africa, including Ghana. Children and adults present with varying clinical and biochemical pictures in response to *Plasmodium* infection. Thus, the study aimed to explore the relationship between *Plasmodium* parasitemia and hematological, liver, and kidney function of children and adult participants, highlighting possible variations.

**Method:**

The cross‐sectional study was conducted in a public and a private health facility in the Eastern Region of Ghana from August 2023 to September 2023. Participants of the study comprised children under 5 years and adults 18 years and above. Structured questionnaires were used to gather participants' demographic information and medical history. Blood samples of participants were analyzed with standard microscopy technique for *Plasmodium* parasites. Hematological and biochemical parameters were also measured with fully automated hematology and chemistry analyzers, respectively. Data were analyzed using IBM SPSS Version 22. *χ* ^2^ analysis was performed to determine if differences in the degree of *Plasmodium* parasitemia and some demographic characteristics were significant. Kruskal–Wallis and post‐hoc pairwise tests were used to determine differences in hematological and biochemical parameters across categories of *Plasmodium* parasitemia. Statistical significance was assumed at a *p* value < 0.05.

**Results:**

The study enrolled 362 participants, comprising of 71.8% (260/362) adults and 69.3% (251/362) females. No significant differences were observed among gender and age categories across parasitemia levels, although a higher proportion of children < 5 years (51.9%, 53/102) recorded high parasitemia. Adult participants with high parasitemia recorded significantly lower Hb, RBC, MCV, MCH and PLT but higher ALT and AST than those with lower and moderate parasitemia. However, there was no significant variations in urea and creatinine levels with regard to degree of parasitemia among the participants.

**Conclusion:**

High *Plasmodium* parasitemia was linked with significant hematological and hepatic alterations, with distinct variations between adults and children < 5. The alterations observed in patients with high parasitemia highlight the importance of frequent and routine monitoring of these patients to address potential dysfunction early to prevent severe complications.

## 1 Introduction

Malaria is characterized by *Plasmodium* parasitemia, the presence of *Plasmodium* parasites in the blood. Most cases of malaria in sub‐Saharan Africa are due to *Plasmodium falciparum*, with *Plasmodium malariae* and *Plasmodium ovale* accounting for a small fraction of cases [1]. Malaria remains a significant public health challenge globally and particularly in sub‐Saharan Africa, including Ghana. In 2021, there were 247 million cases of malaria, up from 245 million cases in 2020 globally. The estimated cases of malaria deaths stood at 619,000 in 2021 compared to 625,000 in 2020 [2]. About 95% of all malaria cases and 96% of all malaria deaths occurred in the WHO African Region, with the most vulnerable age group being children under 5 years old [2]. Ghana has a significant malaria burden; it is one of the top three illnesses treated in outpatient clinics [3]. Over 5.2 million confirmed cases of malaria and 151 deaths related to the disease were reported in Ghana in 2022 [3].

Apart from the classical hematological derangement, including anemia and low platelet count associated with malaria infection, the infection has been reported to affect other organs of the body, such as the liver and the kidneys. There are multiple processes underlying the hematological derangement of the disease. These include splenic sequestration of red cells, reduced erythropoiesis, and the death of parasitised red blood cells [4, 5]. Severe anemia is a frequent and potentially fatal malaria complication among children in endemic regions [6]. Thrombocytopenia is another common hematological disorder linked to malaria [7]. Although the precise causes of thrombocytopenia are unknown, immune‐mediated degradation of platelets and sequestration in the spleen may be involved [8]. Depending on the stage and severity of the infection, patients may experience leukopenia or leukocytosis [9, 10].

One significant side effect of malaria is liver dysfunction, which is caused by the production of inflammatory cytokines and the sequestration of infected erythrocytes in the hepatic microcirculation [11]. Jaundice, elevated blood bilirubin, and changes in liver enzyme levels, including aspartate aminotransferase (AST) and alanine aminotransferase (ALT), are among the clinical signs [12, 13]. Some studies have shown hepatic dysfunction as a common presentation for both adults and children with severe malaria, which can complicate their clinical management [14, 15].

Acute kidney injury (AKI) can result from malarial renal involvement, especially in severe instances [16]. Immune‐mediated injury, severe dehydration and hemolysis‐induced hypovolemia, as well as microvascular blockage by parasitised erythrocytes, are the possible causes of AKI [17]. Some studies have reported a significant elevation in serum creatinine and urea among infected patients compared to uninfected patients, indicating possible renal dysfunction among the infected patients [18, 19].

The normal hematological and biochemical profiles of the liver and kidney differ in children and adults, and *Plasmodium* infection may produce varying hematological and biochemical profiles among adults and children. This assertion is supported by the study by Arévalo‐Herrera et al. [20], where renal dysfunction was more frequent among adults than children with *Plasmodium* infection in Colombia. In the same study, hepatic dysfunction and haemoglobinuria were more frequent in adults than in children. Thus, the above study brings to the fore the importance of the knowledge of the varying hematological and biochemical profiles that may be produced in *Plasmodium* infection among children and adults. The severity of the disease, the response to treatment, and the likelihood of complications may all be impacted by variations in these parameters between children and adults. For example, blood transfusions are frequently required for severe anemia in children who have malaria, whereas adults may be more susceptible to hepatic or renal complications that call for alternative therapeutic approaches [20]. Early diagnosis, risk assessment, and individualized treatment plans can all benefit from the identification of particular patterns of hematological and biochemical changes linked to *Plasmodium* parasitemia. However, there is little information on how malaria‐induced alterations in hematological and biochemical parameters vary between children and adults in Ghana, especially in the Eastern Region. A study by [21], in Atiwa (a district in the Eastern Region) focused on the association between *Plasmodium* infection and some hematological parameters. Our study therefore intended to contribute to the body of knowledge in the field, highlighting the relationship between *Plasmodium* parasitemia and hematological, liver, and kidney function of children and adult participants, underscoring possible variations.

## 2 Method Study Site, Design, and Population

This cross‐sectional study was conducted in the laboratory department of 2 health facilities in the Eastern Region of Ghana, the New Abirem Government Hospital and Medicas Hospital, from August 2023 to September 2023. The New Abirem Government Hospital is a public hospital located at New Abirem, the capital of Birem North Municipality. The Birem North Municipality is located at South‐Western part of the Eastern Region. Medicas is a private hospital located at Mampong in the Akuapim North Municipality in the South‐Eastern part of the Region. These facilities provide general and some specialist care for a wide range of patients. They have diagnostic resources, including laboratories that offer basic laboratory services. By their strategic location on the map of the Eastern Region, South‐Eastern, and South‐Western, the facilities were selected to provide geographic diversity for the study. Also, the public and private hospitals were considered to provide socioeconomic diversity in terms of participant enrollment for the study.

The study population included both male and female children under 5 years of age and adults 18 years and above who presented with a request for malaria investigation at the laboratories of the study sites, and tested positive for malaria parasites.

### 2.1 Sample Size Estimation

The prevalence of clinical malaria among children under 5 years in the Eastern Region was reported to be 6.7% in 2022 by the Ghana Statistical Service Demographic and Health Survey 2022 Report [22]. Also, the prevalence of clinical malaria among adults in the Eastern Region in 2022 was 7.3% according to the 2022 Malaria Parasite Prevalence Report, as reported in the National Malaria Elimination Strategic Plan of Ghana report [3]. Using the Cochran's formula, $N=\frac{Z^{2}(p\;x\;q)}{d²}$, where *N *= sample size; *p *= prevalence of malaria; *q* = 1 − p; *Z *= the critical probability value for a confidence level of 95% (1.96) and *d* = 5% margin of error (0.05), the minimum sample size for the children and adult participants were 96 and 104 respectively. A convenient sampling technique was used to enroll 102 and 260 children and adult participants, respectively.

### 2.2 Inclusion and Exclusion Criteria

The study included participants with clinical suspicion of malaria who were requested by their clinicians to have a blood film for malaria parasite test at the laboratory departments of the study sites, and tested positive for malaria parasites. Pregnant women and participants suspected to have liver diseases, renal disorders, sickle cell disease, chronic anemia, those who smoked, drank alcohol, according to questionnaire information gathered and by observation, were not enrolled in the study. Also, participants who had received malaria treatment in any form, orthodox or herbal, within 3 months of the commencement of the study, were not included. Participants who refused to have their blood samples taken for laboratory investigations were also excluded from the study.

**Figure 1:**
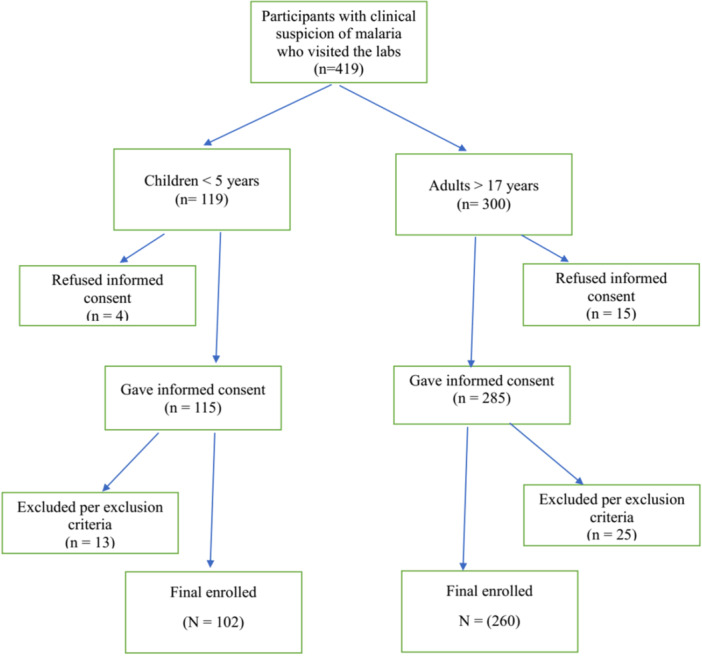
Flow chart of participants' recruitment for the study.

To minimize participant selection bias, the study was conducted in a private and a public hospital to include participants of diverse socioeconomic backgrounds. To avoid the temptation of selecting severely ill participants whose hematological or biochemical parameters could skew data, the study avoided patients on hospital admission and relied on those who visited the laboratory departments. Figure 1 below is a flow chart of how participants were recruited for the study.

### 2.3 Demographic Data Collection

Participants were engaged face‐to‐face with a structured questionnaire after obtaining informed consent from them. The questionnaire was divided into two sections (A and B). Section A assessed the socio‐demographic information of participants. Section B assessed the presence of chronic illnesses; alcohol consumption; smoking; history and symptoms of hematological, liver and kidney disorders; and any treatment/medication received for malaria for the past 3 months.

### 2.4 Blood Sample Collection and Preparation

Five milliliters (5 mL) of venous blood samples were aseptically drawn by venipuncture from the subjects to reduce iatrogenic‐associated infection [23]. About 3 mL of the sample was transferred into a sterile serum separator tube for biochemical analysis, and the remaining 2 mL was dispensed into an ethylene diaminetetraacetic acid (EDTA) tube for hematological analysis [23]. The blood sample in the serum separator tube was left for 30 min to clot at room temperature. Serum was obtained from the clotted sample by centrifugation at 3000 rpm for 5 min. The serum was transferred into a plain tube and used for analysis [24].

### 2.5 Parasitological Investigations

Both thin and thick blood films were prepared on the same glass slide, air‐dried, and stained using 10% Giemsa working solution at a pH of 7.2 for 10 min, with the thin film fixed with methanol before staining [25]. The thin and thick blood films were observed by two trained and qualified malaria microscopists. The slides were declared positive only when the two microscopists concurred. The slides were first scanned with 10× and 40× objective lenses to identify fields for examination and then observed with the 100× objective. At least 100 high‐power fields of the thick film were examined for malaria parasites. The thick smear was labeled “no malaria parasite seen” if no parasites were found following this preliminary screening. White blood cells and asexual parasites were counted simultaneously using two tally counters. Once there were either > 100 parasites in 200 WBCs or < 99 parasites in 500 WBCs, the counting was terminated, and the results were noted appropriately [25]. Thin films were examined to confirm the species identified.

The absolute WBC count from the FBC results and the parasite count were used to determine the parasite density (parasites per microlitre of blood) [25]. *Plasmodium* parasitemia of respondents were then classified as high, moderate and low with corresponding parasite densities of ≥ 10,000, 1000–9999 and < 1000, respectively [26, 27].

### 2.6 Hematological and Biochemical Investigations

The EDTA samples were used for full blood count analysis using a fully automated Mindray BC‐5000 analyzer according to the manufacturer's manual [28]. The analyzer measured the hematological parameters by the following techniques: white blood cell (WBC)—flow cytometry by laser method; hemoglobin (Hb)—colorimetric method; red blood cell (RBC) and platelet (PLT)—electrical impedance method; mean cell volume (MCV)—calculated from RBC histogram; and MCH—calculated from Hb and RBC [28]. The analyzer was well calibrated, and each batch of test run included controls. External quality control assessments were periodically done with control samples from reference laboratories in other regions of Ghana.

The serum samples were measured for liver function test (LFT) markers such as total protein (TP), albumin (ALB), total bilirubin (TB), aspartate aminotransferase (AST), alanine aminotransferase (ALT), alkaline phosphatase (ALP), and kidney function markers (urea and creatinine) using a fully automated chemistry analyzer (RAYTO Chemray 120). The analyzer operates with the specific turbidity transmission method and calculates the concentration of samples according to the Beer–Lambert Law [29]. The biomarkers were measured by the following International Federation of Clinical Chemistry and Laboratory Medicine (IFCC) analytical methods using Fortress Diagnostics clinical chemistry reagent kits [30]): AST and ALT—kinetic ultraviolet (UV) enzymatic assay; ALP—*p*‐nitrophenyl phosphate (pNPP) kinetic method; Urea– urease enzymatic UV kinetic method; Creatinine—modified Jaffe reaction; TB—Diazo reaction; ALB—Bromocresol Green (BCG) dye‐binding method; TP—Biuret colorimetric method. Every test batch run included controls, and the assay was performed in accordance with the manufacturer's instructions for the analyzer and reagents [29].

### 2.7 Statistical Analysis

The data obtained from the laboratory, as well as the questionnaires, were well documented and transferred onto a Microsoft Excel spreadsheet. The data were then imported and analyzed with the IBM SPSS Version 22 statistical tool. Frequencies, percentages and means were calculated. For these, results were illustrated as frequency tables. *χ* ^2^ analysis was used to determine if differences in the degree of *Plasmodium* parasitemia and some demographic characteristics were significant. Continuous variables with skewed distributions were summarized using median and interquartile range (IQR). Differences in hematological and biochemical parameters across parasitemia categories were assessed using the Kruskal–Wallis test and the accompanying post‐hoc pairwise test. Statistical significance was assumed at *p* values < 0.05 in a two‐tailed manner.

### 2.8 Ethical Consideration

Ethical clearance for the study was obtained from Koforidua Technical University (KTU) Ethical Review Board with reference number KTU/DRE/ERB/MLSB/28. Permission to conduct the study at the health facilities was sought from the heads of laboratories and administrators of the facilities. Informed consent was obtained from participants before they were enrolled in the study. For children, informed consent was obtained from their parents. Participants were given unique identification codes written on the study consent form, questionnaire and sample bottles. The filled consent forms, questionnaires and laboratory results are stored under lock and key in a secure location at KTU. All listed authors met the International Committee of Medical Journal Editors (ICMJE) criteria.

## 3 Results

### 3.1 Demographic Characteristics of Respondents

The study enrolled 362 participants, comprising of 71.8% adults and 28.2% children under 5 years. The majority of the respondents were females (69.3%). Most of the female (75.3%) and male (64.0%) respondents were adults. Table 1 shows the demographic characteristics of respondents.

**Table 1:** Demographic characteristics of respondents.

Demographic characteristics			*N* = 362	Percentage
Age	< 5 years		102	28.2
	≥ 18 years		260	71.8
Sex	Female		251	69.3
	Male		111	30.7
Sex by age	Female	< 5 years	62	24.7
		≥ 18 years	189	75.3
	Male	< 5	40	36.0
		≥ 18 years	71	64.0

### 3.2 Classification of *Plasmodium* Parasitemia Among Respondents

*Plasmodium falciparum* was the only *Plasmodium* species identified in the blood samples of the participants. Although not statistically significant, a higher proportion of children < 5 years (51.9%) recorded high parasitemia than adults (42.3%). Also, a higher proportion of females (12.40%) recorded low parasitemia than males (10.80%), but there was no difference between females and males with regard to high parasitemia. Table 2 shows the degree of *Plasmodium* parasitemia among respondents.

**Table 2:** *χ* ^2^ Analysis of *Plasmodium* parasitemia among respondents.

		Classification of parasitemia		Low *n* (%)	*χ* ^2^	*p* value
		High *n* (%)	Moderate *n* (%)			
Age	< 5 years	53 (51.9%)	42 (41.2%)	7 (6.9%)	4.645	0.098
	> 17 years	110 (42.3%)	114 (43.80%)	36 (13.9%)		
Sex	Female	113 (45.0%)	107 (42.6%)	31 (12.4%)	0.195	0.907
	Male	50 (45.0%)	49 (44.2%)	12 (10.8%)		

*Note:* Percentages across rows sum up to 100%, *χ* ^2^ = Chi‐square value.

### 3.3 Classification of *Plasmodium* Parasitemia and the Hematological, Liver, and Kidney Functions of Respondents

The results show a significant association between parasitemia level and differences in hematological and liver function among respondents. In general, high parasitemia was associated with relatively lower Hb, RBC, MCH, and PLT count but higher ALT and AST than all other levels of parasitemia (Tables 3a and 3b). Also, high parasitemia was associated with higher WBC and ALP than moderate parasitemia levels. However, respondents recorded no significant differences in urea and creatinine levels per the degree of *Plasmodium* parasitemia (Tables 3a and 3b).

**Table 3a:** Kruskal–Wallis test of the distribution of hematological and biochemical parameters across *Plasmodium* parasitemia levels of respondents.

Parameters		Classification of *Plasmodium* parasitemia			*p* value
		High *n* = 163	Moderate *n* = 156	Low *n* = 43	
		Median (IQR)	Median (IQR)	Median (IQR)	
Hematological	Hb (g/dL)	10.3 (4.2)	11.6 (3.7)	11.9 (2.3)	**< 0.001** *
	RBC (10^6^/µL)	3.9 (1.4)	4.2 (1.2)	4.9 (1.1)	**< 0.001** *
	MCV (fL)	82.9 (8.7)	83.3 (9.6)	86.6 (9.7)	**0.014** *
	MCH (pg)	27.2 (3.3)	27.4 (3.4)	28.1 (3.0)	**0.004** *
	WBC (10^3^/µL)	7.3 (4.6)	6.2 (3.7)	7.1 (3.5)	**0.043** *
	PLT (10^3^/µL)	144.0 (113.0)	169.0 (111.8)	212.0 (112.0)	**< 0.001** *
Biochemical	ALT (U/L)	23.6 (25.0)	20.3 (15.5)	18.4 (24.3)	**0.004** *
	AST (U/L)	25.0 (18.4)	18.9 (12.4)	18.8 (13.8)	**0.003** *
	ALP (U/L)	88.4 (81.9)	63.7 (81.9)	88.0 (95.5)	**0.019** *
	TP (g/L)	69.0 (9.6)	69.8 (11.7)	70.0 (11.6)	0.224
	ALB (g/L)	38.2 (14.0)	38.2 (7.5)	38.5 (8.4)	0.611
	GLO (g/L)	28.4 (6.1)	26.5 (6.3)	26.4 (7.1)	0.084
	TB (µmol/L)	14.7 (6.2)	14.5 (5.9)	15.8 (7.0)	0.137
	UREA (mmol/L)	4.8 (3.2)	4.3 (2.7)	4.9 (1.9)	0.080
	CREA (µmol/L)	91.8 (24.0)	91.5 (18.3)	91.1 (20.6)	0.402

*Note:* Bold values indicate statistically significant.

Abbreviations: ALB = albumin, ALP = Alkaline phosphatase, ALT = alanine aminotransferase, AST = aspartate aminotransferase, CREA = creatinine, GLO = globulin, Hb = hemoglobin, IQR = interquartile range, MCH = mean cell hemoglobin, MCV = mean cell volume, PLT = platelet, RBC = red blood cell, TB = total bilirubin, TP = total protein, WBC = white blood cell.

* *p* value < 0.05.

**Table 3b:** Post‐hoc pairwise comparisons of significant Kruskal–Wallis test variables of respondents.

Variable	Classification of parasitemia (1)	Classification of parasitemia (2)	Test statistic	*p* value
Hb (g/dL)	High	Low	71.308	**< 0.001** *
		Moderate	45.846	**< 0.001** *
RBC (10^6^/µL)	High	Low	66.317	**< 0.001** *
		Moderate	37.690	**0.001** *
MCV (fL)	High	Low	49.337	**0.006** *
		Moderate	21.586	0.066
MCH (pg)	High	Low	51.826	**0.004** *
		Moderate	28.808	**0.014** *
WBC (10^3^/µL)	High	Low	5.339	0.766
		Moderate	28.780	**0.014** *
PLT (10^3^/µL)	High	Low	71.173	**< 0.001** *
		Moderate	36.637	**0.002** *
ALT (U/L)	High	Low	40.667	**0.023** *
		Moderate	35.130	**0.003** *
AST (U/L)	High	Low	40.593	**0.024** *
		Moderate	36.111	**0.002** *
ALP (U/L)	High	Low	21.722	0.211
		Moderate	32.870	**0.005** *

*Note:* Bold values indicate statistically significant.

Abbreviations: ALP = alkaline phosphatase, ALT = alanine aminotransferase, AST = aspartate aminotransferase, CREA = creatinine, Hb = hemoglobin, MCH = mean cell hemoglobin, PLT = platelet, RBC = red blood cell, TB = total bilirubin, WBC = white blood cell.

* *p* value < 0.05.

### 3.4 Classification of *Plasmodium* Parasitemia and Hematological, Liver, and Kidney Function of Adult Respondents

Among adult participants, higher parasitemia was significantly associated with lower Hb, RBC and PLT but higher ALT and AST than all other levels of parasitemia. Higher parasitemia was also associated with lower MCV and MCH than low parasitemia (Tables 4a and 4b). However, there were no significant differences in urea and creatinine values with regard to parasitemia levels (Tables 4a and 4b).

**Table 4a:** Kruskal–Wallis test of the distribution of hematological and biochemical parameters across *Plasmodium* parasitemia levels of adult respondents.

Parameters		Classification of *Plasmodium* Parasitemia			*p* value
		High *n* = 110	Moderate *n* = 114	Low *n* = 36	
		Median (IQR)	Median (IQR)	Median (IQR)	
Hematological	Hb (g/dL)	9.9 (4.4)	11.8 (3.8)	11.9 (2.6)	**< 0.001** *
	RBC (10^6^/µL)	3.7 (1.5)	4.4 (1.2)	4.4 (1.1)	**< 0.001** *
	MCV (fL)	83.7 (9.4)	83.4 (8.7)	87.6 (7.7)	**0.043** *
	MCH (pg)	27.3 (3.4)	27.5 (3.2)	28.5 (3.1)	**0.007** *
	WBC (10^3^/µL)	7.2 (4.4)	6.2 (3.3)	7.0 (3.6)	0.141
	PLT (10^3^/µL)	150.0 (106.5)	170.5 (105.0)	202.0 (71.0)	**0.011** *
Biochemical	ALT (U/L)	23.5 (24.4)	20.0 (13.1)	18.7 (20.1)	**0.007** *
	AST (U/L)	25.6 (8.3)	19.8 (12.5)	18.7 (13.3)	**0.011** *
	ALP (U/L)	84.6 (73.1)	73.6 (85.6)	88.7 (94.4)	0.251
	TP (g/L)	69.4 (13.6)	69.6 (10.6)	69.9 (11.8)	0.385
	ALB (g/L)	38.3 (15.1)	38.2 (7.3)	38.9 (8.7)	0.879
	GLO (g/L)	28.9 (5.9)	26.8 (5.7)	26.9 (6.4)	0.245
	TB (µmol/L)	14.3 (6.3)	14.9 (5.4)	15.8 (7.4)	0.517
	UREA(mmol/l)	4.8 (3.3)	4.4 (2.7)	4.8 (2.2)	0.326
	CREA(µmol/L)	93.0 (28.7)	9.7 (18.9)	91.4 (21.6)	0.240

*Note:* Bold values indicate statistically significant.

Abbreviations: ALB = albumin, ALP = alkaline phosphatase, ALT = alanine aminotransferase, AST = aspartate aminotransferase, CREA = creatinine, GLO = globulin, Hb = hemoglobin, IQR = interquartile range, MCV = mean cell volume, MCH = mean cell hemoglobin, PLT = platelet, RBC = red blood cell, TB = total bilirubin, TP = total protein, WBC = white blood cell.

* *p* value < 0.05.

**Table 4b:** Post‐hoc pairwise test of significant Kruskal–Wallis test variables of adult respondents.

Variable	Classification of parasitemia (1)	Classification of parasitemia (2)	Test statistic	*p* value
Hb (g/dL)	High	Low	53.253	**< 0.001** *
		Moderate	43.311	**< 0.001** *
RBC (10^6^/µL)	High	Low	53.603	**< 0.001** *
		Moderate	40.007	**< 0.001** *
MCV (fL)	High	Low	36.236	**0.012** *
		Moderate	8.576	0.394
MCH (pg)	High	Low	44.203	**0.002** *
		Moderate	18.023	0.073
PLT (10^3^/µL)	High	Low	40.776	**0.005** *
		Moderate	19.810	**0.049** *
ALT (U/L)	High	Low	29.971	**0.038** *
		Moderate	29.401	**0.003** *
		Low	35.778	**0.013** *
AST	High	Moderate	24.560	**0.015** *

*Note:* Bold values indicate statistically significant.

Abbreviations: ALP = alkaline phosphatase, ALT = alanine aminotransferase, AST = aspartate aminotransferase, CREA = creatinine, Hb = hemoglobin, MCH = mean cell hemoglobin, PLT = platelet, RBC = red blood cell, TB = total bilirubin, WBC = white blood cell.

* *p* value < 0.05.

### 3.5 Classification of *Plasmodium* Parasitemia and Hematological, Liver, and Kidney Function of Children

In children < 5, high parasitemia was associated with significantly lower PLT count than all other categories of parasitemia. It was also associated with higher ALP but had no significant association with the kidney function of respondents (Tables 5a and 5b).

**Table 5a:** Kruskal–Wallis test of the distribution of hematological and biochemical parameters across *Plasmodium* parasitemia levels of children respondents.

Parameters		Classification of *Plasmodium* parasitemia			*p* value
		High *n* = 53	Moderate *n* = 42	Low *n* = 7	
		Median (IQR)	Median (IQR)	Median (IQR)	
Hematological	Hb (g/dL)	10.8 (3.4)	10.9 (3.7)	11.0 (4.2)	0.159
	RBC (10^6^/µL)	4.1 (1.2)	4.1 (1.2)	4.8 (1.8)	0.221
	MCV (fL)	79.6 (9.4)	82.0 (15.5)	79.1 (21.8)	0.333
	MCH (pg)	26.9 (3.2)	27.3 (3.9)	25.7 (5.1)	0.185
	WBC (10^3^/µL)	8.0 (7.1)	6.4 (4.8)	7.5 (7.8)	0.282
	PLT (10^3^/µL)	120.0 (118.0)	160.5 (197.0)	270.0 (142.0)	**0.001** *
Biochemical	ALT (U/L)	25.1 (27.1)	22.9 (18.9)	17.9 (34.6)	0.580
	AST (U/L)	22.2 (15.3)	18.0 (6.8)	20.7 (35.0)	0.144
	ALP (U/L)	99.0 (90.8)	59.4 (82.0)	79.2 (105.0)	**0.040** *
	TP (g/L)	68.7 (7.0)	72.3 (11.9)	70.5 (10.0)	0.288
	ALB (g/L)	37.9 (9.5)	37.9 (7.7)	37.9 (7.0)	0.496
	GLO (g/L)	28.0 (6.4)	26.0 (5.9)	24.6 (4.9)	0.143
	TB (µmol/L)	15.4 (6.9)	13.8 (8.7)	15.4 (4.8)	0.126
	UREA(mmol/l)	4.3 (3.1)	3.9 (2.3)	5.3 (1.1)	0.058
	CREA(µmol/L)	91.6 (17.8)	89.8 (16.7)	89.8 (20.0)	0.823

*Note:* Bold values indicate statistically significant.

Abbreviations: ALB = albumin, ALT = alanine aminotransferase, ALP = alkaline phosphatase, AST = aspartate aminotransferase, CREA = creatinine, GLO = globulin, Hb = hemoglobin, IQR = interquartile range, MCH = mean cell hemoglobin, MCV = mean cell volume, PLT = platelet, RBC = red blood cell, TB = total bilirubin, TP = total protein, WBC = white blood cell.

* *p* value < 0.05.

**Table 5b:** Post‐hoc pairwise test of significant Kruskal–Wallis test variables of children respondents.

Variable	Classification of parasitemia (1)	Classification of parasitemia (2)	Test statistic	*p* value
PLT (10^3^/µL)	High	Low	36.230	**0.002** *
		Moderate	16.552	**0.007** *
ALP (U/L)	High	Low	14.508	0.223
		Moderate	14.949	**0.014** *

*Note:* Bold values indicate statistically significant.

Abbreviations: ALP = alkaline phosphatase, PLT = platelet.

* *p* value < 0.05.

## 4 Discussion

The study investigated the relationship between *Plasmodium* parasitemia and hematological, liver and kidney function of participants. The findings indicate that a higher proportion of children < 5 years recorded high parasitemia than adults. This finding is corroborated by the study conducted by [31], where children in malaria‐endemic areas in Ghana had a higher rate of parasitemia than adults. The immune system of children, especially those < 5 years, is still developing, which makes them more vulnerable to malaria infection [32]. Adults usually have more exposure to malaria than children do, as the immune response to the disease develops with repeated exposure [33]. Children's heightened vulnerability is exacerbated by socioeconomic and environmental problems, which are common in low‐income countries, including Ghana. Low maternal educational attainment, and living in both rural and impoverished families are some socio‐economic factors implicated in the high rates of malaria in children under five [34, 35].

High parasitemia predisposes patients to severe malaria and increase risk of mortality. Younger children are at higher risk of high parasitemia and severe *falciparum* malaria, as indicated by WHO [36] and this study. Children with severe *falciparum* malaria commonly experience complications like cerebral malaria, severe anemia, respiratory distress (acidosis) and hypoglycemic more than adults [36]. Therefore, the holistic management of children with high parasitemia and severe malaria varies from adults, although both are required to promptly start intravenous or intramuscular anti‐malaria medications [36].

The study revealed no difference between males and females with regard to high parasitemia. This finding is in tune with a study conducted in Ghana, where there was no significant difference between males and females in terms of parasite density, despite recording more females [37]. However, some studies have reported gender differences in *Plasmodium falciparum* infection clearance and prevalence, suggesting biological differences in malaria infection dynamics [38, 39]. But these findings do not necessarily translate to differences in parasite density between sexes.

The findings of the study highlight the diverse effects of Plasmodium parasitemia on the hematological, renal, and hepatic systems of the body. Relatively lower Hb, RBC, MCV, MCH, and PLT were observed among participants with high parasitemia than those with other parasitemia levels. This result is in line with other recent research that shows comparable patterns among malaria patients [40, 41]. A study by Njewa, Eyong, and Ebai [42], suggested possible anemia and thrombocytopenia by showing a substantial correlation between high parasitemia and a decrease in Hb, RBC and PLT counts, highlighting the critical need for monitoring and managing these parameters to prevent severe clinical outcomes. A higher WBC count was recorded among respondents with high parasitemia, reflecting an immune response to the infection, [43] reported similar findings, where increased WBC count was observed in patients with high parasitemia, indicating the important role of the immune system in parasitic infections.

Generally, relatively higher ALT, AST and ALP were observed among participants with high parasitemia, which may indicate liver involvement and possible hepatic stress. The finding is in line with the study by [44], which discovered that individuals with high parasitemia showed substantial elevations in liver enzymes, suggesting possible hepatic injury or stress. To prevent and treat potential hepatic injury, our data emphasize the importance of liver function monitoring in individuals with high parasitemia. However, the study found no significant relationship between urea and creatinine levels and the degree of *Plasmodium* parasitemia among the general study population and adult and children subpopulations. A similar trend was observed in Nigeria, where urea and creatinine levels had no significant relationship with varying parasite densities among adults [18, 45] also reported weak or no correlation (*r* = 0.17, *p* = 0.05) between creatinine levels and peripheral parasitemia among adult patients with severe *falciparum* malaria in Bangladesh. Again in Nigeria [46], found no significant changes in creatinine and varying parasitemia levels among young children infected with *Plasmodium falciparum*. These results suggest that parasite density in isolation may not reliably predict alteration in kidney biomarkers and kidney dysfunction in *falciparum* malaria. Thus, we suggest monitoring of kidney function in *falciparum* malaria regardless of parasite load.

The study found varying significant relationships between *Plasmodium* parasitemia and hematological and liver function of adults and children respondents. Among adult participants, high parasitemia was associated with relatively lower Hb, RBC, MCH, and PLT but higher ALT and AST. Whereas among children < 5 years, high parasitemia was linked with relatively lower PLT but higher ALP. A similar trend was observed among adult and children population in other part of the Eastern Region, where a negative correlation was observed between parasite density and levels of Hb and PLT [21, 42], also reported a strong correlation between high parasitemia and decreased platelet counts in young children, suggesting a heightened risk of severe disease outcomes. With regard to liver biomarkers [12], reported significant elevations in AST and ALT levels among adult patients with high parasitemia. Whereas [47], observed increased ALP levels among children with high parasitemia, suggesting possible liver involvement or stress. These revelations highlight the importance of routine investigations of FBC and LFT in the management of both adult and children patients with high parasitemia.

The findings of the study should be considered against the background of some limitations. The study excluded subjects with comorbidities that could affect biochemical and hematological parameters directly and indirectly, with a questionnaire and not by laboratory investigations, due to resource constraints. Laboratory screening for comorbidities is thus strongly suggested in future studies for enhanced exclusion criteria. The cross‐sectional nature of the study limits causality interpretation; we suggest prospective or longitudinal follow‐up in future studies. Additionally, the convenience sampling method adopted may have introduced participants' selection bias, limiting the generalizability of results. Random sampling is proposed in future studies.

## 5 Conclusion

In conclusion, high parasitemia was linked with significant hematological and hepatic alterations with variations between adults and children < 5, underscoring the systemic impact of *Plasmodium* infections. The observed hematological alterations indicate that routine FBC is necessary to identify and treat anemia and thrombocytopenia in patients with high parasitemia as soon as possible. Additionally, the alterations in liver biomarkers observed among participants with high parasitemia indicate that routine LFT investigation is important to address potential hepatic dysfunction early. No variations were observed in renal function of respondents with regard to the degree of parasitemia, suggesting that the renal function of patients be monitored regardless of parasite load. Monitoring of hematological, liver and kidney biomarkers is vital for the holistic management of *Plasmodium* infections and can help prevent severe complications. Future research is suggested to throw more light on the underlying mechanisms of high parasitemia and alterations in hematological, renal and liver function, and to develop targeted interventions to improve patient outcomes.

## Author Contributions

Conceptualization by A.B, D. TM., R.A.A, and V.K.O. Analysis by A.B, D.TM, M.H.A, R.A.A, and V.K.O. Methodology by A.B, I.B, K.M, M.A, and A.S.K. Project administration by D.TM, R.A.A, V.K.O., and C.Y. Writing of original draft, review and editing: A.B, I.B, K.M, M.A, C.Y, and A.S.K.

## Funding

The authors received no specific funding for this work.

## Conflicts of Interest

The authors declare no conflicts of interest.

## Transparency Statement

The corresponding author, Tonnies Abeku Buckman, affirms that this manuscript is an honest, accurate, and transparent account of the study being reported; that no important aspects of the study have been omitted; and that any discrepancies from the study as planned (and, if relevant, registered) have been explained.

## Data Availability

Data available upon reasonable request to the corresponding author with appropriate ethical approval.
